# Experimental investigation on modes of spray formation, droplet size and size distribution in a spinning disc atomizer

**DOI:** 10.3389/fpls.2024.1470745

**Published:** 2024-12-05

**Authors:** Jian Chen, Wei Hu, Xiaoya Dong, Jinlong Lin, Zhouming Gao, Baijing Qiu

**Affiliations:** ^1^ School of Agricultural Engineering, Jiangsu University, Zhenjiang, China; ^2^ Key Laboratory of Plant Protection Equipment, Ministry of Agriculture and Rural Affairs, Jiangsu University, Zhenjiang, China; ^3^ Key Laboratory of Modern Agricultural Equipment and Technology, Ministry of Education, Jiangsu University, Zhenjiang, China; ^4^ Key Laboratory of Modern Agricultural Equipment of Jiangxi, Jiangxi Agricultural University, Nanchang, China

**Keywords:** spinning disc atomizer, spray formation mode, droplet size, droplet size distribution, Rosin-Rammler distribution

## Abstract

The spinning disc atomizer is extensively utilized in agricultural spraying, with optimized operating conditions significantly enhancing atomization performance. In this paper, the atomization characteristics of a spinning disc were studied using photographs taken by a high-speed camera. Ethanol-water solutions were used at various flow rates and the disc speed was varied in a wide range. The influence of disc speed, flow rate, and surface tension on modes of spray formation, droplet size, and size distribution were investigated. The correlations for Reynolds number (*Re*), Stability number (*St*), and dimensionless droplet size (d^*^) were proposed in a wide range of operational conditions. The Rosin-Rammler (RR) and modified Rosin-Rammler (MRR) distributions appropriately represented the droplet size distribution. It was found that the increase in flow rate resulted in modes of spray formation translation under the same disc speed and ethanol-water solution. The predicted droplet sizes showed good agreement with the experiment values. Most of the predicted droplet sizes were within the band of ±15% of the experiment values. The droplet size decreased with increasing *Re* or *St*, but was hardly affected by *q*. Besides, the droplet size decreased with increasing disc speed and decreasing surface tension. The RR and MRR distribution matched with the calculated cumulative volume fraction from the experimental data reasonably well for the entire range. It was recommended to appropriately elevate *Re* during the spinning disc atomization process to narrow the range of droplet sizes and enhance uniformity.

## 1 Introduction

Rotary atomizers, characterized by a narrow droplet size distribution and an open structure that is resistant to clogging, have significant advantages over hydraulic nozzles. Hence, they are extensively used in many applications, such as spray pesticides (Ru et al., 2024; Liu et al., 2024), spray coating (Gödeke et al., 2021; Sidawi et al., 2021), and slag granulation (Mantripragada et al., 2021; Li et al., 2023).

In these rotary atomizers, the liquid is directly fed into the center of a spinning disc, cup, or cage. The liquid is forced to the edge by centrifugal forces, then ejected from the edge, and broken into small droplets (Peng et al., 2017; Sahoo and Kumar, 2024). Hinze and Milborn (1950) identified three different modes of spray formation. As the flow rate increases, the modes of spray formation will transition from direct drop to ligament formation, and then further to sheet formation. Frost (1981) confirmed this phenomenon and highlighted the presence of mixed modes of spray formation. He established dimensionless correlations for the transition of spray formation modes. Glahn et al. (2002); Peng et al. (2016), and Wu et al. (2018) also developed correlations for the transition of spray formation modes on a spinning disc. The correlations from these studies show significant variances, likely due to differences in operational conditions and atomizer configurations (Feng et al., 2021).

To better understand the factors affecting droplet size in spinning disc atomization, various research teams have conducted numerous experiments. Walton and Prewett (1949) estimated the droplet size by impaction of the droplets on magnesium oxide coated slides and measurement of the crater diameters. They found that spinning disc atomization can produce uniform droplet sprays and revealed the relationship between average droplet size, disc speed, surface tension, and density. Boize and Dombrowski (1976) used sub-microsecond spark photography to study the atomization characteristics of oils with different viscosities under various operating conditions. They found that droplet size is mainly influenced by disc speed. Frost (1981) measured droplet size using short-duration spark photography and developed a droplet size correlation. This further revealed the relationship between droplet size and various influencing factors. Ahmed and Youssef (2012) measured droplet sizes under different operating conditions using a phase-doppler particle analyzer. The results indicated that droplet size is related to various dimensionless numbers. Wang et al. (2016) used a high-speed camera to experimentally study the ligament formation and break-up process at the edge of the spinning disc. They established the relationship between average droplet size, disc speed, and liquid physical properties. Kumar and Sarkar (2019) investigated the impact of slotted discs on droplet size, while Prather et al. (2023) analyzed the atomization dynamics and their effect on droplet size in the spinning disc atomization process. **Table 1** summarizes the correlations of droplet size with spinning disc atomizers from previous studies, listing the test liquids, range of operating variables, and modes of spray formation. However, the correlations established by these studies are not consistent. The differences are mainly due to variations in droplet size measurement techniques, atomizer structures, and modes of spray formation (Prather et al., 2023; Ahmed and Youssef, 2012). Additionally, different definitions of droplet size may also lead to varying results. The correlation of droplet size is based on droplet distribution corresponding to droplet diameters, including arithmetic mean diameter (*D*_10_), Sauter mean diameter (*D*_32_), and volume median diameter (*D*_V0.5_ or VMD) (ASTM E799-03(2020)e1, 2020; Wang et al., 2022). Although these parameters provide measurements of droplet size, *D*_V0.5_ is usually used in agricultural spray applications to better evaluate the overall size distribution (Sijs et al., 2021).

**Table 1:** Correlations of droplet size by spinning disc atomizers.

Authors	Liquids	Correlations	Modes of formation	Range of variables
(Walton and Prewett, 1949)	Water, mercury, methyl salicylate	$D_{10}=3.8{\left(\frac{\sigma }{\rho D\omega^{2}}\right)}^{0.5}$	Direct drop, ligament, and sheet	$\begin{array}{l} Q=2.4-168\text{ ml}\cdot {\text{min}}^{-1}\\ \omega =477-95493 r\cdot {\text{min}}^{-1}\\ D=0.02-0.08\text{ m}\\ \mu =0.001-1.5\text{ Pa}\cdot \text{s}\\ \rho =900-1360\text{ kg}\cdot {\text{m}}^{-3}\\ \sigma =31-465\text{ mN}\cdot {\text{m}}^{-1} \end{array}$
(Boize and Dombrowski, 1976)	Oils	$D_{V0.5}=0.0107\omega^{-1.09}$ $D_{32}=0.006\omega^{-0.98}$	Direct drop and ligament	$\begin{array}{l} Q=7.5-240\text{ ml}\cdot {\text{min}}^{-1}\\ \omega =750-6000\text{ r}\cdot {\text{min}}^{-1}\\ D=0.088\text{ m}\\ \mu =0.0073-0.0603\text{ Pa}\cdot \text{s}\\ \rho =834-868\text{ kg}\cdot {\text{m}}^{-3}\\ \sigma =28-35\text{ mN}\cdot {\text{m}}^{-1} \end{array}$
(Frost, 1981)	Aqueous solutions of glycerol and proprietary wetting agent	$D_{V0.5}=1.87\frac{Q^{0.44}\sigma^{0.15}\mu^{0.017}}{D^{0.80}\omega^{0.75}\rho^{0.16}}$	Ligament	$\begin{array}{l} Q=60-600\text{ ml}\cdot {\text{min}}^{-1}\\ \omega =477-9549 r\cdot {\text{min}}^{-1}\\ D=0.04-0.12\text{ m}\\ \mu =0.001-0.022\text{ Pa}\cdot \text{s}\\ \rho =1000-1170\text{ kg}\cdot {\text{m}}^{-3}\\ \sigma =33-59\text{ mN}\cdot {\text{m}}^{-1} \end{array}$
(Ahmed and Youssef, 2012)	Water	$\begin{array}{l} \frac{D_{32}}{D}=27.81{\left[\frac{Q}{\omega D^{3}}\right]}^{0.051}{\left[\frac{R}{D}\right]}^{0.581}\\ \;{\left[\frac{D^{2}\omega \rho }{\mu }\right]}^{-0.651}{\left[\frac{D^{3}\omega^{2}\rho }{\sigma }\right]}^{-0.0218} \end{array}$	Ligament	$\begin{array}{l} Q=33.36-166.8\text{ ml}\cdot {\text{min}}^{-1}\\ \omega =8002-16014 r\cdot {\text{min}}^{-1}\\ D=0.04-0.12\text{ m}\\ \mu =0.001-0.022\text{ Pa}\cdot \text{s}\\ \rho =1000-1170\text{ kg}\cdot {\text{m}}^{-3}\\ \sigma =33-59\text{ mN}\cdot {\text{m}}^{-1} \end{array}$
(Wang et al., 2016)	glycerol/water mixture	$\frac{D_{10}}{R}=2.582We^{-0.321}Oh^{0.251}{\left(\frac{\rho Q^{2}}{\sigma R^{3}}\right)}^{1/7}$	Ligament	$\begin{array}{l} Q=0-1800\text{ ml}\cdot {\text{min}}^{-1}\\ \omega =600-3000 r\cdot {\text{min}}^{-1}\\ D=0.1\text{ m}\\ \mu =0.00528-0.0175\text{ Pa}\cdot \text{s}\\ \rho =1113-1170\text{ kg}\cdot {\text{m}}^{-3}\\ \sigma =73-74.3\text{ mN}\cdot {\text{m}}^{-1} \end{array}$
(Kumar and Sarkar, 2019)	Water	D10R=0.48We−0.137Re0.14Oh0.25	Direct drop and ligament	$\begin{array}{l} Q=211.8-924\text{ ml}\cdot {\text{min}}^{-1}\\ \omega =200-1200\text{ r}\cdot {\text{min}}^{-1}\\ D=0.15\text{ m}\\ \mu =0.001\text{ Pa}\cdot \text{s}\\ \rho =1000\text{ kg}\cdot {\text{m}}^{-3}\\ \sigma =72\text{ mN}\cdot {\text{m}}^{-1} \end{array}$
(Prather et al., 2023)	Compritol 888	$D_{10}=\sqrt{\frac{6\sigma }{\rho r\omega^{2}}}+51$ $\begin{array}{l} D_{32}=1.1\times 1.6R\\ \;\left(\frac{4\rho Q^{0.26}}{\pi \mu R}{\frac{\rho \omega^{2}R^{3}}{\sigma }}^{-0.42}{\frac{\mu }{\sqrt{\rho \sigma R}}}^{0.38}\right)+17 \end{array}$	Direct drop and ligament	$\begin{array}{l} Q=80-280\text{ ml}\cdot {\text{min}}^{-1}\\ \omega =1000-2500 r\cdot {\text{min}}^{-1}\\ D=0.1016\text{ m}\\ \mu =0.018\text{ Pa}\cdot \text{s}\\ \rho =863\text{ kg}\cdot {\text{m}}^{-3}\\ \sigma =21.1\text{ mN}\cdot {\text{m}}^{-1} \end{array}$

In agricultural spray applications, droplet size distribution is a critical factor in determining spraying efficacy, directly impacting coverage, drift risk, and deposition and absorption on target plants (Gong et al., 2022b, 2022a). Droplet size distribution is typically presented as histograms and cumulative distribution curves. Boize and Dombrowski (1976) analyzed droplet size distribution across various liquid viscosities, disc speeds, and flow rates using histograms. Frost (1981) illustrated the uniformity of droplet size distribution using cumulative distribution curves. To more accurately evaluate droplet size distribution, researchers have employed the VMD-NMD (Number median diameter, NMD) ratio, coeficient of variation, and relative span factor (RSF) as assessment metrics. Panneton (2002) assessed the uniformity of droplet size distribution generated by spinning cups under various geometric parameters and operating conditions using the VMD-NMD ratio, and explored key influencing factors. Sahoo and Kumar (2021a) systematically studied the size distribution of primary and secondary droplets in the direct droplet mode of a spinning disc and quantified their coefficient of variation. Ru et al. (2024) employed the RSF to investigate the factors influencing droplet size distribution in rotary nozzles. Additionally, the practical and mathematically simple Rosin-Rammler (RR) distribution has been applied to droplet size distribution in rotating packed beds (Xie et al., 2022; Gao et al., 2015) and rotary-bell atomizers (Corbeels et al., 1992). However, compared to these applications, the droplet size distribution of spinning disc atomizers in agricultural sprays is broader and contains more large droplets, warranting further investigation.

This study used ethanol-water solutions as the test liquids and utilized a high-speed camera to analyze the atomization characteristics of a spinning disc atomizer. The research investigated the effects of disc speed, flow rate, and surface tension on the modes of spray formation, droplet size, and size distribution. Based on the experimental results, a dimensionless correlation for droplet size was established, and the statistical distribution of droplet sizes around *D_V0.5_* was analyzed.

## 2 Materials and methods

### 2.1 Experimental setup

**Figure 1:**
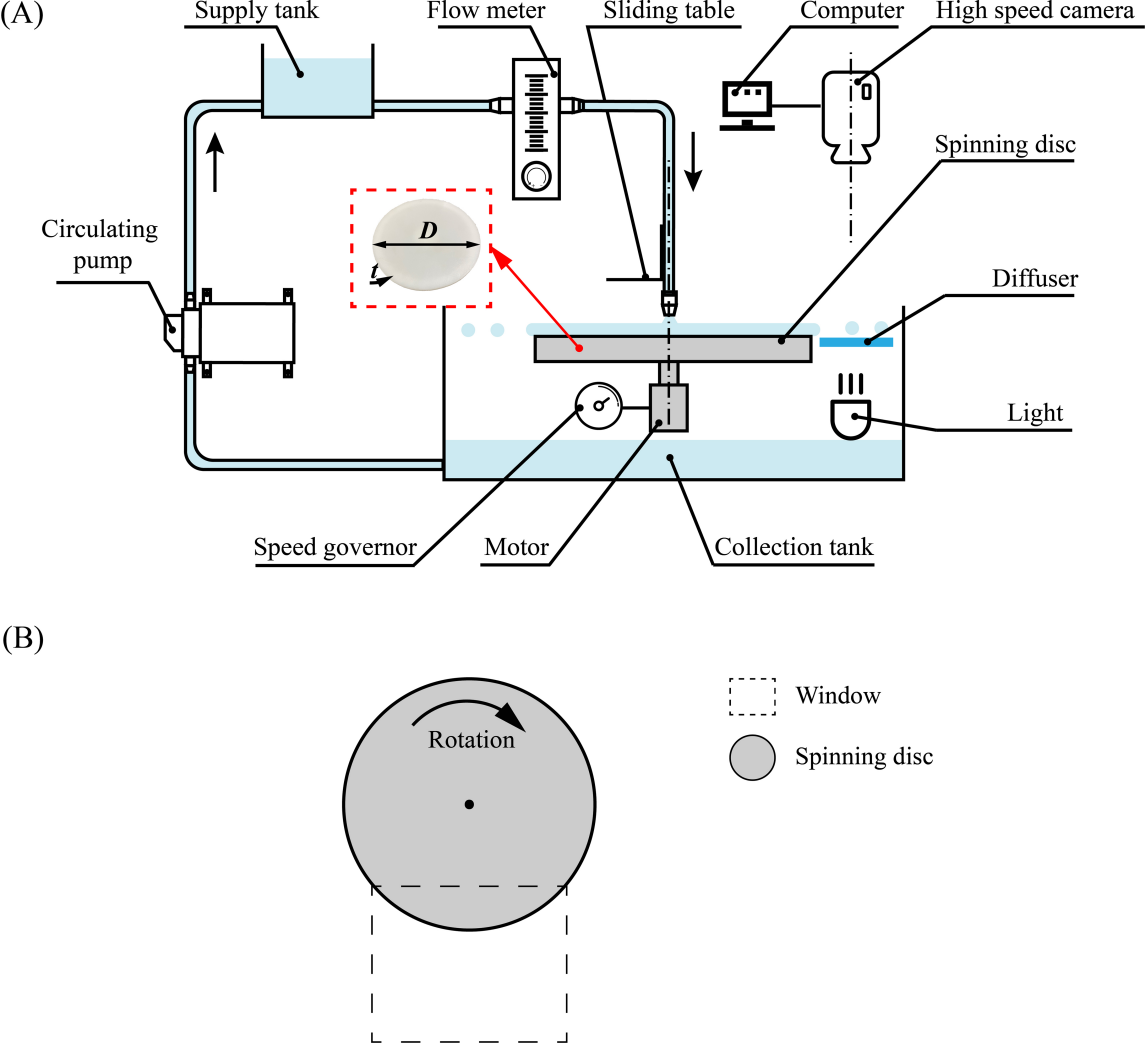
Schematic diagram of **(A)** experimental setup for spinning disc atomization and **(B)** image acquisition windows (top view). The diameter of the disc is *D* = 0.06 m and the thickness is *t* = 0.003 m.

The experimental setup consisted of a spinning disc atomizer, a feed system, and a visualization system (**Figure 1A**). The disc was made of photopolymer resin and polished with fine sandpaper to remove burrs before spraying. The disc had a diameter of 0.06 m and an edge thickness of 0.003 m, and was driven by a stepper motor. The rotational speed was adjusted by a speed governor and varied between 1000 and 4000 r·min^-1^ in increments of 500 r·min^-1^. The liquid was delivered to the disc by gravity through a 6 mm diameter circular nozzle from the supply tank. The nozzle was positioned vertically above the disc at a distance of 5 mm from the surface. The liquid volumetric flow rate was adjusted by a flow meter on the supply tube. The droplets were collected in a collection tank and returned to the supply tank by a circulating pump. A high-speed camera (SH6-109, Shenzhen Sincevision Technology Co., Ltd, China) was installed above the disc, with a panel light source positioned below it. **Figure 1B** presents a top view illustrating the relative positions of the camera window and the disc.

### 2.2 Test liquids

To vary the surface tension of the spray liquid without significantly altering the viscosity, various volume percentages of ethanol-water solutions were used, following Kooij et al. (2018) (**Table 2**). The surface tension varied with different volume percentages and was measured using the Wilhelmy plate method with a Krüss Force Tensiometer K100. The density was measured using a pycnometer, with each measurement repeated three times. In the experiments, changes in ethanol concentration had minimal effect on viscosity (Yao and Fang, 2013; Kooij et al., 2018). Therefore, the viscosity was approximated to be equal to that of water, which is 0.001 Pa·s.

**Table 2:** Surface tension and density of ethanol-water solutions.

Vol %ethanol	Surface tension (mN·m^-1^)	Density (kg·m^-3^)
0	72.535 ± 0.072	997.9 ± 0.4
9	51.764 ± 0.083	985.6 ± 1.5
50	29.681 ± 0.025	930.1 ± 0.1

### 2.3 Experimental operating conditions

During measurements, the ambient temperature was maintained at approximately 20°C. Five flow rates, three concentrations of ethanol-water solutions, and seven disc speeds were used, resulting in 105 operating points. For capturing images of droplet size measurements, the flow rate varied between 100 and 500 ml·min^-1^ in increments of 100 ml·min^-1^. To further capture the space of spray formation modes, additional flow rates (50, 600, and 880 ml·min^-1^) were tested with a 50% ethanol-water solution. At the operating point, 9500 frames per second were captured at a resolution of 1280 × 1024. To prevent image blurring caused by high-speed rotation, the camera exposure time was set to a low value of 5 μs. Prior to experimentation, images of a 1.5 mm × 1.5 mm checkerboard were captured to determine spatial resolution. Image acquisition commenced approximately 15 seconds after spray initiation, once a stable spray state was achieved. To avoid capturing the same droplet multiple times, images were acquired in intervals: after each pair of images, a 60-frame interval was imposed before capturing the subsequent pair. A minimum of 1088 image pairs were recorded for each operating point. Matlab software (R2019b, MathWorks, Natick, MA, USA) was employed to determine the size and size distribution of droplets in the image. To ensure data reliability, over 2000 droplets were analyzed for each operating point. Each operation was repeated three times, maintaining a coefficient of variation below 7.5%.

### 2.4 Dimensional analysis

In the study of spinning disc atomization, the relationships among various variables were complex. To simplify the relationships among variables and better understand their effects on *D*_V0.5_, dimensional analysis was employed. The density of the surrounding air is negligible compared to the liquid density, thus it is not considered a major variable. Consequently, the *D*_V0.5_ of spinning disc atomization depends solely on the liquid’s physical properties (density *ρ*, viscosity *μ*, surface tension *σ*), disc diameter (*D*), and operating conditions (disc speed *ω*, flow rate *Q*). Thus, the studied physical phenomena are summarized in the following relationship:


(1) $$D_{V0.5}=f(\omega ,\;\sigma ,\;Q,\;\rho ,\;\mu ,\;D)$$

According to Buckingham’s π theorem, four dimensionless quantities were derived (Buckingham, 1914):


(2) $$\frac{D_{V0.5}}{D}=F(\frac{D^{2}\omega \rho }{\mu },\;\frac{2\mu^{2}}{\rho \sigma D},\;\frac{\rho Q}{D\mu })$$

Where: [*D*_V0.5_ */D*] is defined as dimensionless droplet size, d^*^; [*D* ^2^ *ωρ*/*μ*] is defined as the Reynolds number, *Re*, representing the ratio of inertial forces to viscous forces; [2*μ* ^2^/*ρσD*] is defined as the Stability number, *St*, which is proportional to the ratio of viscous to inertial and surface tension forces (Bizjan et al., 2014; Li et al., 2022); [*ρQ*/*Dμ*] is defined as the dimensionless flow rate, *q*.

A widely accepted monomial form was employed for correlating d* as a function of *Re*, *St*, and *q*. The application of the monomial form to Equation 2 gives Equation 3:


(3) d*=CReαStβqγ

### 2.5 Droplet size distribution functions

To make useful predictions, it is essential to consider the statistical distribution of droplet sizes around the characteristic diameter. Among the various options, the RR distribution function is the most commonly used in droplet breakup studies (Lefebvre and McDonell, 2017; Johansen et al., 2013). The RR distribution is a two-parameter function defined by the characteristic diameter *d_i_*, corresponding to a specific cumulative volume fraction, and the spread parameter *n*. The cumulative volume distribution function is:


(4) $$V(d)=1-\exp\left(-k_{i}{\left(\frac{d}{d_{i}}\right)}^{n}\right)$$

where *k_i_* = -ln(1-*V_i_*), for *V_i_* = 50%, *d_i_* represents the median diameter, and *k_i_* = -ln(0.5) =0.693. The exponent *n* indicates a measure of the spread of drop sizes, with higher values of *n* signifying a more uniform spray distribution.


(5) $$V(d)=1-\exp\left(\ln(0.5){\left(\frac{d}{D_{V0.5}}\right)}^{n}\right)$$

Rizk and Lefebvre (1985) proposed a Modified Rosin-Rammler (MRR) distribution function to better fit over most of the drop size range, as shown in the following equation:


(6) $$V(d)=1-\exp\left(\ln(0.5){\left(\frac{\ln(d)}{\ln(D_{V0.5})}\right)}^{n}\right)$$

## 3 Results and discussion

### 3.1 Modes of spray formation

#### 3.1.1 Evolution of modes of spray formation

**Figure 2:**
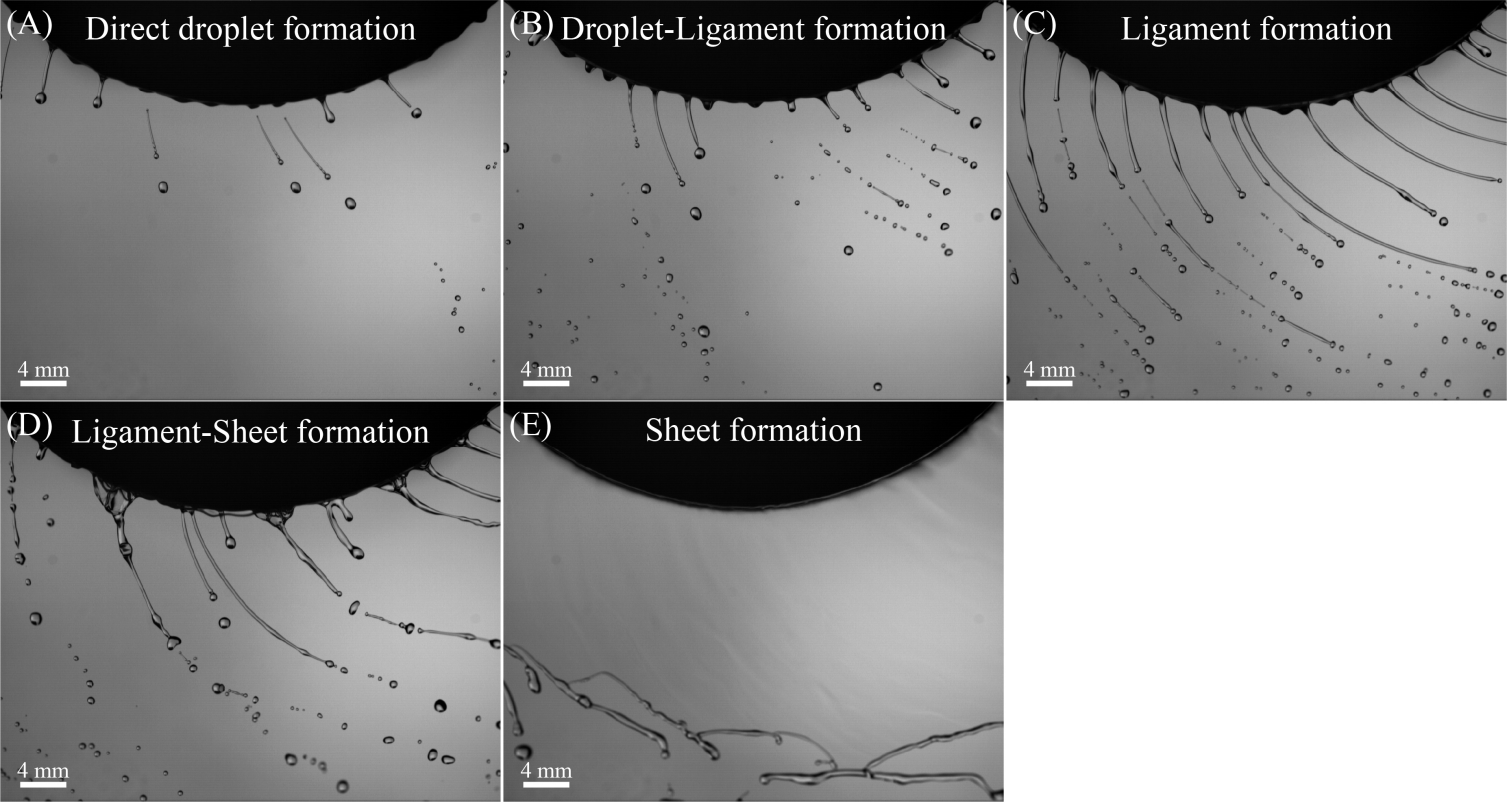
Modes of spray formation of 50% ethanol-water solutions in spinning disc atomization process. **(A)** Direct droplet formation (50 ml·min^-1^ and 1000 r·min^-1^); **(B)** Droplet-Ligament formation (200 ml·min^-1^ and 1000 r·min^-1^); **(C)** Ligament formation (500 ml·min^-1^ and 1000 r·min^-1^); **(D)** Ligament-Sheet formation (600 ml·min^-1^ and 1000 r·min^-1^); **(E)** Sheet formation (880 ml·min^-1^ and 1000 r·min^-1^).

**Figure 2** illustrates the transition in modes of spray formation as the flow rate increases, with the spinning disc operating at 1000 r·min^-1^ and using a constant ethanol-water solution. At a low flow rate of 50 ml·min^-1^, direct droplet formation is initially observed at the edge of the disc (**Figure 2A**). Driven by centrifugal force, the liquid spreads outward, forming a radial liquid film on the disc surface. Once the liquid film reaches the disc’s edge, it extends a finite distance radially, forming a liquid torus around the edge (Wang et al., 2015; Tan et al., 2019). Rayleigh-Taylor instability, induced by centripetal acceleration, destabilizes the torus, forming one or more bulges (Keshavarz et al., 2020; Eisenklam, 1964). Primary droplets form by elongating the bulge, followed by the creation of smaller satellite droplets from the liquid thread connecting the primary droplet with the bulge (Sahoo and Kumar, 2021a, 2021b). This mode is commonly employed in handheld and aerial rotary atomizers.

If the disc is oversupplied with liquid, this direct droplet formation mode is disrupted. When the flow rate increases to 200 ml·min^-1^, a mixture of direct droplet formation and ligament formation is observed (**Figure 2B**). At this stage, ligaments are drawn out from the disc edge, but their distribution is chaotic and disordered. Some droplets still form directly from the disc edge, while others are formed from the breakup of ligaments (Peng et al., 2017). As the flow rate increases to 500 ml·min^-1^, the mode transitions to ligament formation, with droplets formed directly being completely replaced by those formed from ligaments. Distinct, regular ligaments form with stable, orderly flight trajectories (**Figure 2C**). The ligaments break into droplets due to Plateau–Rayleigh instability, with disturbances of wavelength larger than the ligament diameter causing instability, eventually breaking up the ligaments and forming droplets (Rayleigh, 1879). This mode typically results in the formation of uniform droplets proportional to the disturbance wavelength (Li et al., 2018).

When the flow rate reaches 600 ml·min^-1^, adjacent ligaments begin to merge, forming a liquid sheet (**Figure 2D**). At a flow rate of 880 ml·min^-1^, the liquid film on the disc thickens, resulting in fully developed sheet formation (**Figure 2E**). In this mode, the liquid film leaves the disc as a free sheet and then breaks up irregularly (Prather et al., 2023; Li et al., 2018). Although the droplets formed in this mode are less uniform than those from ligament formation, overall, droplets from these three modes are more uniform than those produced by hydraulic nozzles (Salah et al., 2014; Yang et al., 2023).

#### 3.1.2 Space of modes of spray formation

Frost (1981) studied spinning disc atomization using aqueous solutions of glycerol and Agral surfactant. He established transition correlations for spray formation modes through dimensional analysis, see **Appendix A**.

**Figure 3:**
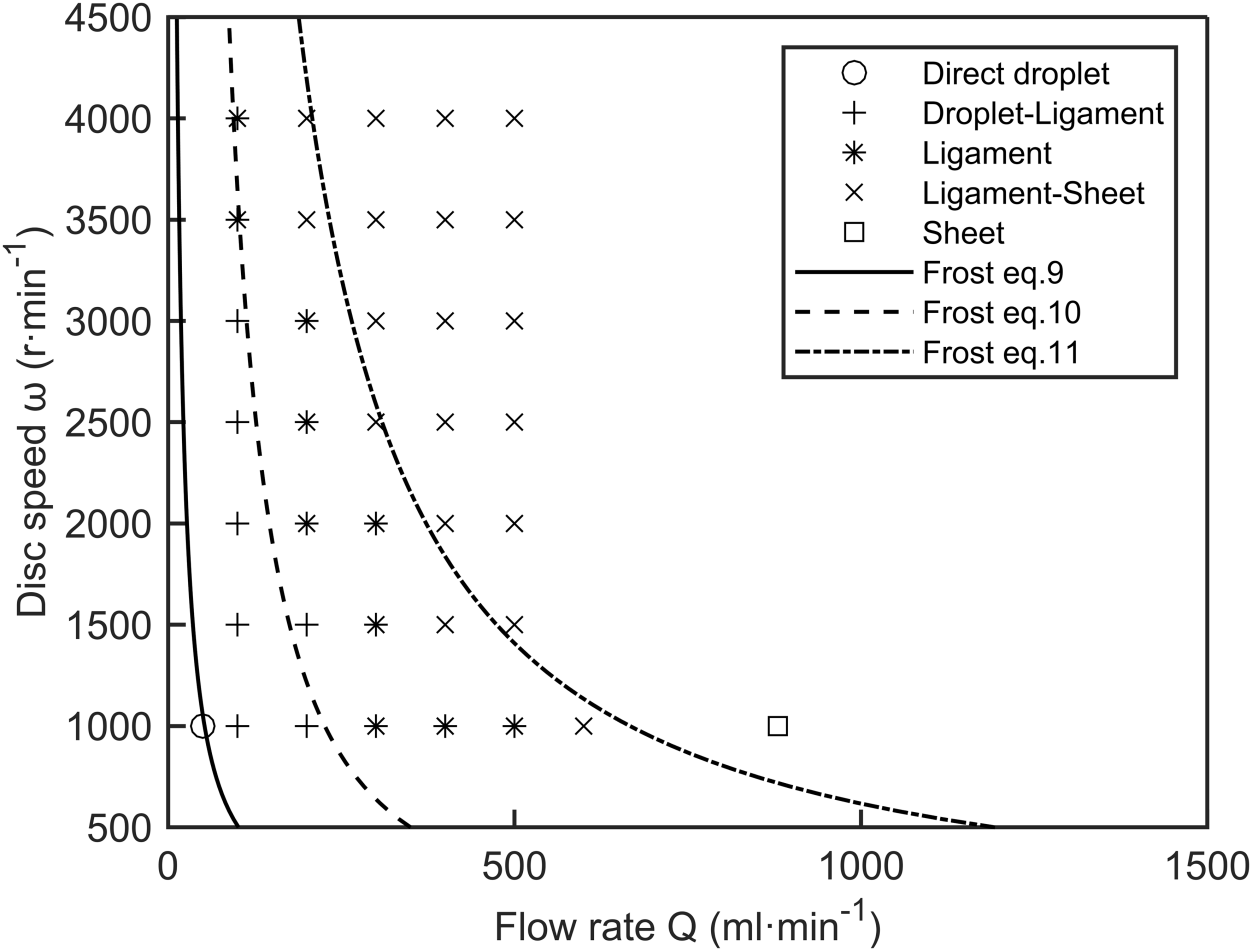
Space of spray formation modes, as predicted by Frost, overlaid with experimental results of 50% ethanol-water solution. Circles represent direct droplet formation, plus signs represent droplet-ligament formation, asterisks represent ligament formation, crosses represent ligament-sheet formation, and squares represent sheet formation.

**Figure 3** presents the spray formation modes predicted by Frost’s transition correlations (9)-(11), with different symbols indicating various operating points. These modes were identified through visual inspection of recorded images and are depicted by the features shown in **Figure 2**. Overall, the predicted spray formation modes closely matched the experimental results, except for ligament formation and ligament-sheet formation. The transition from ligament to sheet formation occurred earlier than predicted, indicating a more abrupt change. This discrepancy may result from subjectivity in defining the mode boundaries or because the equilibrium surface tension used in Frost’s calculations was lower than the actual surface tension. Frost (1981) used aqueous solutions of glycerol and Agral surfactant as test liquids, where the surface tension of the liquid after forming a new surface was equivalent to that of the glycerol solution. Due to the slow molecular dynamics of Agral surfactant, its delivery rate to the droplet surface is insufficient to reach equilibrium, causing the actual surface tension to be higher than the equilibrium surface tension (Kooij et al., 2018; Varghese et al., 2024).

### 3.2 Droplet size analysis and correlations

#### 3.2.1 Correlation for droplet size

*D*_V0.5_ represents the droplet diameter at which half of the spray volume consists of droplets smaller than this size, and the other half consists of droplets larger than this size (Xiang et al., 2021). Frost (1981) proposed a correlation to predict the droplet size produced by a spinning disc atomizer, expressed as:


(7) $$D_{V0.5}=1.87\frac{Q^{0.44}\sigma^{0.15}\mu^{0.017}}{D^{0.80}\omega^{0.75}\rho^{0.16}}$$

**Figure 4:**
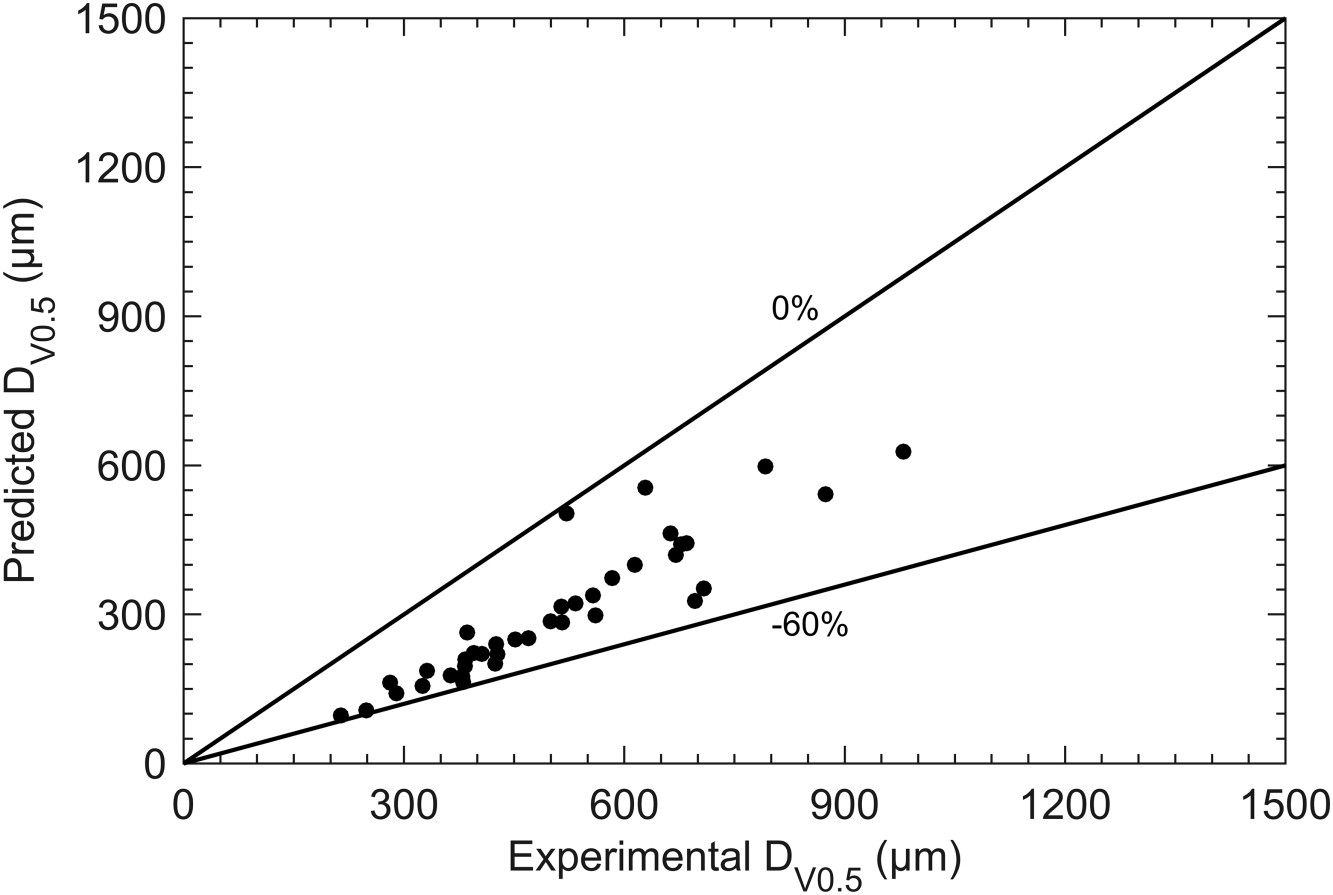
Comparison between measurement data (ligament formation mode) and Frost’s correlation (7) predicted data.

Under the ligament formation mode, **Figure 4** shows that the *D*_V0.5_ predicted by Frost’s correlation is significantly lower than the experimental results, with an error range from 0% to -60%. This underestimation may be due to the equilibrium surface tension used in Frost’s correlation being lower than the actual surface tension, as previously discussed. Therefore, to improve prediction accuracy, it is preferable to use test liquids with faster molecular dynamics when establishing the correlation.

**Figure 5:**
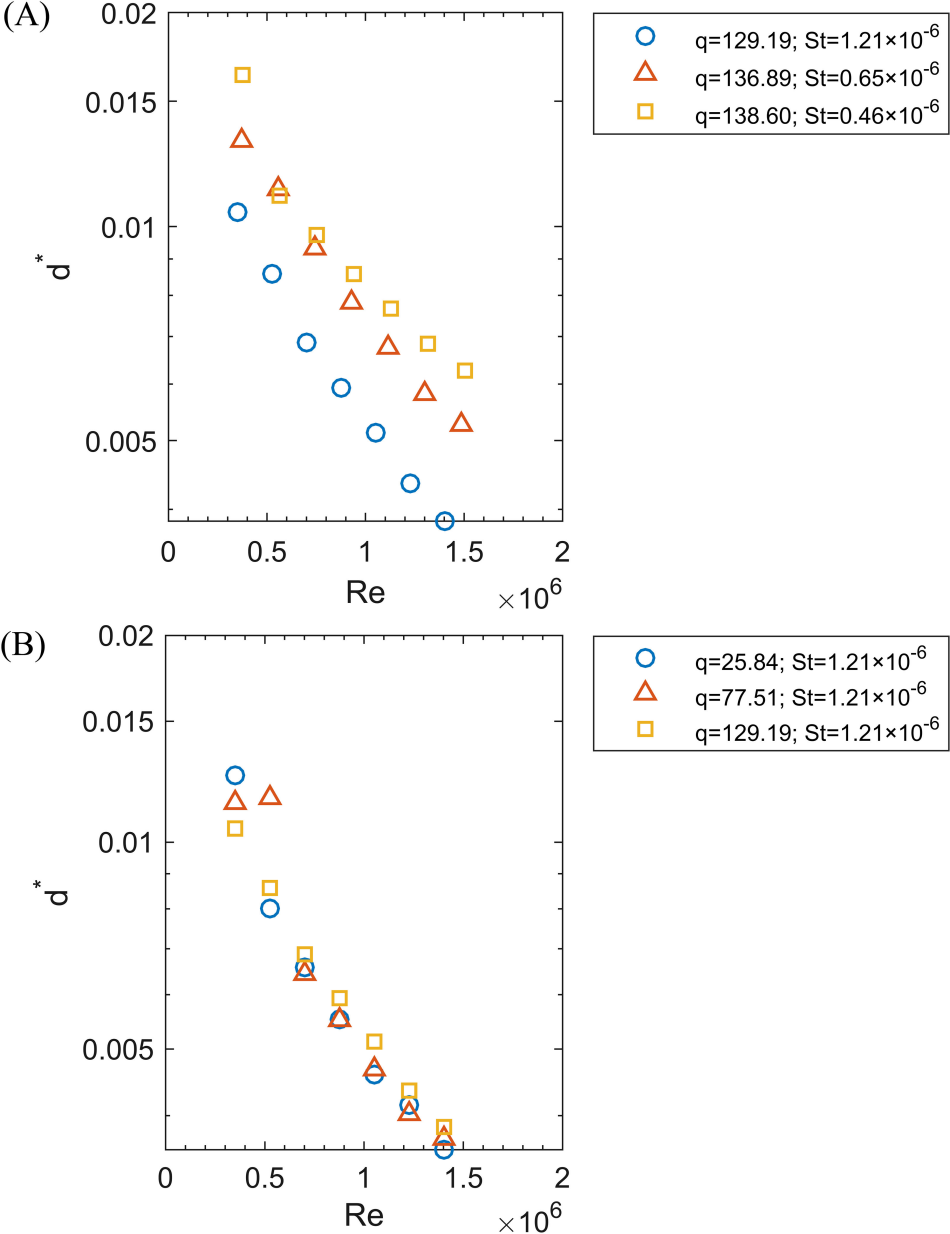
The relationship between *Re* (0.35×106 to 1.50×106) and dimensionless droplet size (d*). **(A)** At a nearly constant q (129.19 to 138.60), St varies from 0.46×10-6 to 1.21×10-6; **(B)** At St = 1.21×10-6, q varies from 25.84 to 129.19.

**Figure 5** illustrates that as the *Re* increased from 0.35×10^6^ to 1.50×10^6^, reflecting a rise in disc speed, the d* decreased across all conditions. In **Figure 5A**, with a nearly constant *q* ranging from 129.19 to 138.60, an increase in *St* from 0.46×10^-6^ to 1.21×10^-6^, reflecting a decrease in surface tension, led to a reduction in d*. Additionally, at *St* = 1.21×10^-6^, **Figure 5B** demonstrates that the *q* (from 25.84 to 129.19) associated with flow rate had a negligible effect on d*.

The *Re* values range from 0.35×10^6^ to 1.50×10^6^, *St* values range from 0.46×10^-6^ to 1.21×10^-6^, and *q* values range from 25.84 to 138.60. The correlation was first fitted according to the Equation 3. However, the *p*-value for the coefficient γ associated with *q* is 0.108, being higher than 0.05, indicating this variable is statistically insignificant. This finding aligned with the results shown in **Figure 5B**. Therefore, *q* was removed from the dimensionless parameters of interest. By analyzing the coefficients in the Equation 3, a dimensionless droplet size correlation was obtained:


(8) $$d^{*}=0.277Re^{-0.814}St^{-0.530}\;(R^{2}=0.951)$$

**Figure 6:**
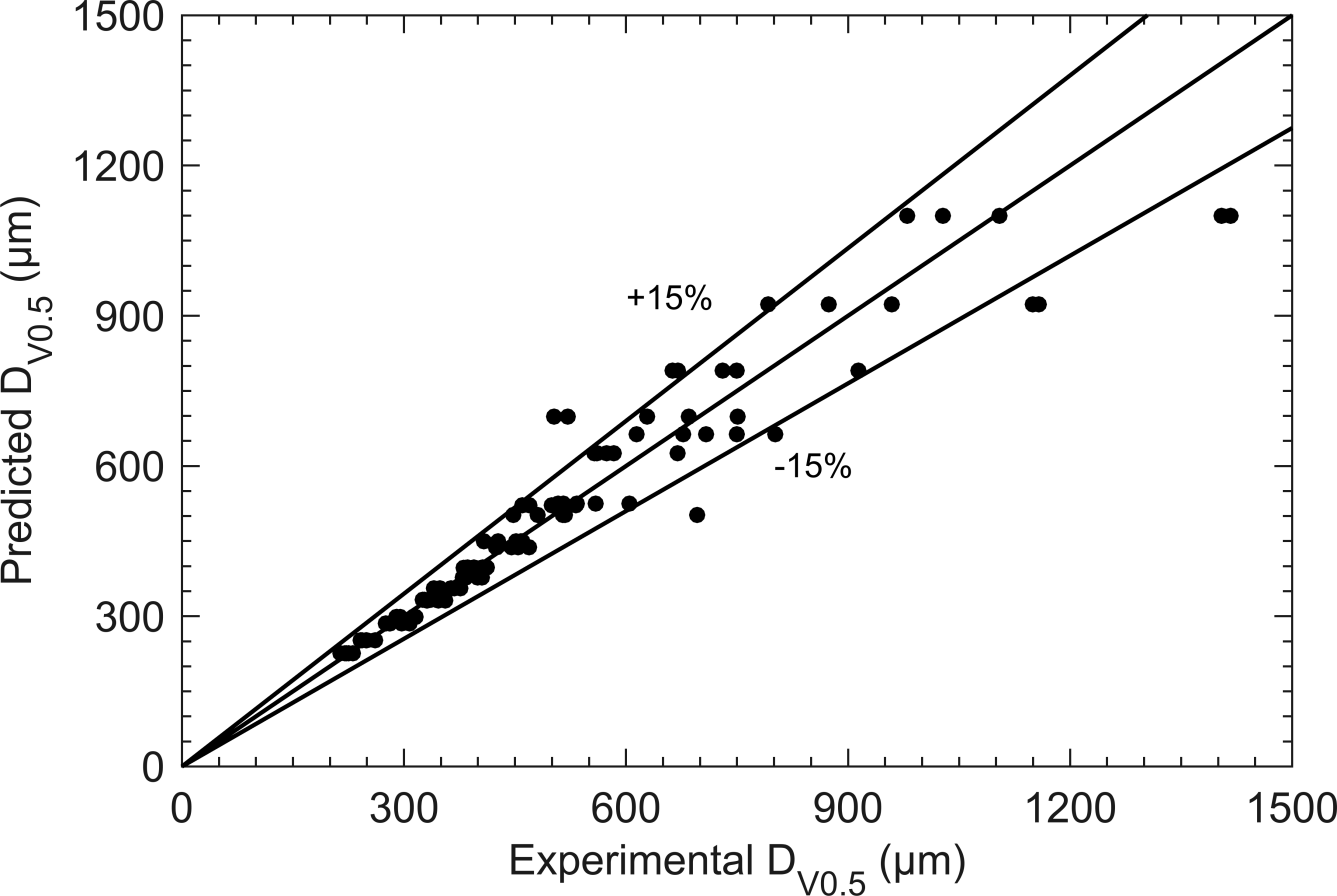
Comparison between measurement data and correlation (8) predicted data.

The magnitude of the coefficient associated with each dimensionless number gives some essential guidance on the phenomena’s prevalence in the spinning disc atomization process. The coefficient associated with *Re* and *St* in the Equation 8 corroborated the conclusions presented in **Figure 5**. **Figure 6** presents a comparison of experimental and predicted droplet sizes. The results indicate that the relative error between most experimental and predicted data is within ±15%, demonstrating the high accuracy and reliability of the Equation 8. This consistency also indicates the high regularity of the spinning disc atomization process. However, some larger droplet sizes exceeded the ±15% error range. This discrepancy may result from poor droplet uniformity at these operating points, affecting the measured *D*_V0.5_ values.

Although this study was conducted in a simplified laboratory environment without considering field complexities such as wind speed, temperature, and humidity, which can indeed influence atomization efficiency and pesticide deposition in practical applications (Lin et al., 2021), the droplet size correlation proposed here holds substantial value for droplet size control. According to the theory of biological optimum droplet size, droplets of specific sizes are more likely to adhere to target organisms, thus enhancing pesticide adhesion and efficacy (Uk, 1977; Chen et al., 2022). Consequently, by adjusting disc speed or liquid surface tension under the guidance of the droplet size correlation, droplets can be generated that align with the biological optimum droplet size, thereby improving pest control effectiveness.

#### 3.2.2 Effect of disc speed on droplet size

**Figure 7:**
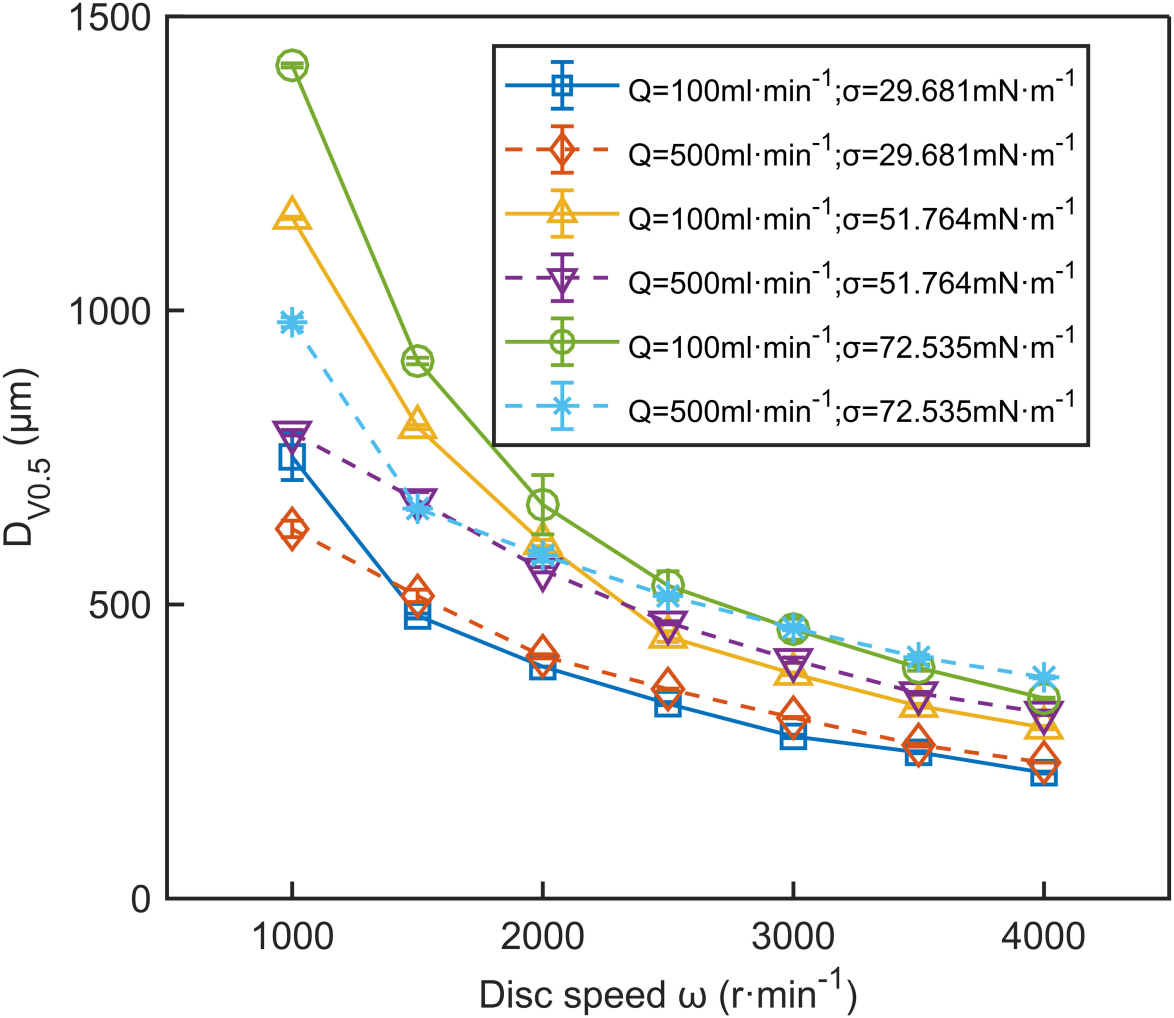
The effect of disc speed on *D*_V0.5_. The data in the figure are the average values from three replications. Error bars represent the standard errors of the means.

Under all operating conditions, *D*_V0.5_ decreased as disc speed increased. **Figure 7** illustrates the relationship between disc speed and *D*_V0.5_. The blue solid line in **Figure 7** indicates that at a flow rate of 100 ml·min^-1^ and surface tension of 29.681 mN·m^-1^, *D*_V0.5_ decreases from 751 μm to 214 μm as disc speed increases from 1000 r·min^-1^ to 4000 r·min^-1^, a total reduction of 537 μm. Between 1000 r·min^-1^ and 2500 r·min^-1^, *D*_V0.5_ decreased by 420 μm, accounting for 78.2% of the total reduction. This reduction may be due to the increased centrifugal force at higher speeds, which pushes the liquid to the disc edge, thins the liquid film, and forms smaller droplets (Mantripragada and Sarkar, 2017; Ahmed and Youssef, 2012). Moreover, Wu et al. (2018) noted that stronger shear effects within the liquid film and at the liquid-solid and gas-liquid interfaces contribute to smaller droplet sizes. However, as disc speed further increases, the slip velocity between the liquid film and the disc surface increases, slowing the reduction of liquid film thickness, which leads to a plateau in the decrease of *D*_V0.5_ at higher disc speeds (Peng et al., 2018a).

#### 3.2.3 Effect of flow rate on droplet size

**Figure 8:**
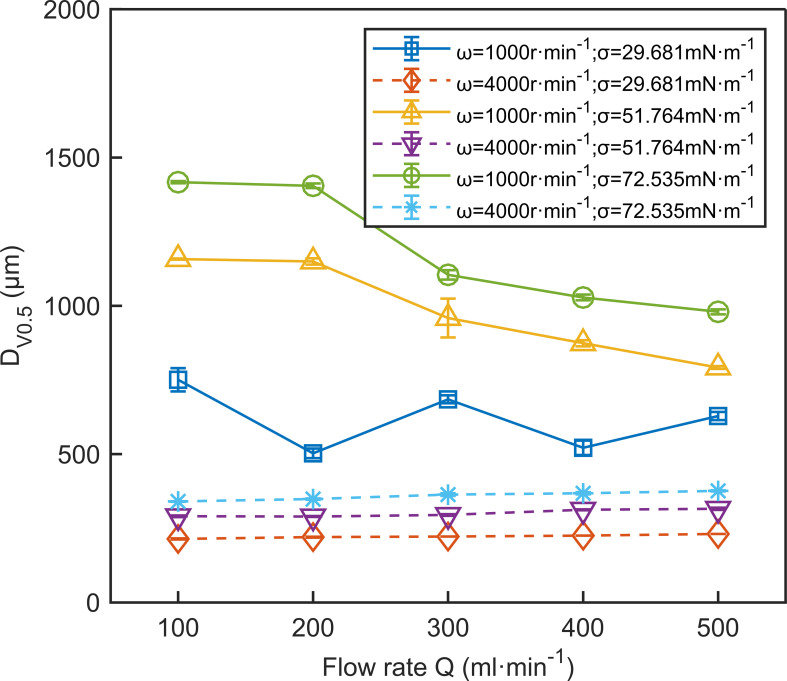
The effect of flow rate on *D*_V0.5_. The data in the figure are the average values from three replications. Error bars represent the standard errors of the means.

The flow rate influences droplet size, but its effect is less significant than that of disc speed. **Figure 8** depicts the effect of flow rate on *D*_V0.5_. The solid line in **Figure 8** shows that at low disc speeds, *D*_V0.5_ heavily depends on the flow rate. At high disc speeds, as indicated by the dashed line, this dependence decreases significantly. At high disc speeds, *D*_V0.5_ slightly increases with the rising flow rate. For instance, at a disc speed of 4000 r·min^-1^, the orange dashed line in **Figure 8** shows that as the flow rate increases from 100 ml·min^-1^ to 500 ml·min^-1^, *D*_V0.5_ increases from 214 μm to 231 μm. This could be due to the spray formation mode showing more liquid ligament characteristics, where long ligaments extend from the disc edge. An increase in flow rate may lead to more liquid ligaments or their elongation, thus accommodating the increased liquid volume (Wang et al., 2016; Peng et al., 2018b). As shown in **Figure 7**, when the disc speed exceeds 2500 r·min^-1^, *D*_V0.5_ is primarily controlled by disc speed, and the effect of flow rate becomes negligible. This phenomenon is supported by the study of the Micron ULVA rotary atomizer by Boize and Dombrowski (1976). At low disc speeds, *D*_V0.5_ changes significantly with an increasing flow rate. For instance, the goldenrod solid line in **Figure 8** shows that at a disc speed of 1000 r·min^-1^ and a surface tension of 51.764 mN·m^-1^, increasing the flow rate decreases droplet size. As the flow rate increases from 100 ml·min^-1^ to 500 ml·min^-1^, *D*_V0.5_ decreases from 1158 μm to 792 μm. This might be due to a change in spray formation mode caused by the increase in flow rate. In the ligament formation mode, the droplet sizes produced are much smaller than those in the direct droplet mode at the same disc speed (Prather et al., 2023).

#### 3.2.4 Effect of surface tension on droplet size

**Figure 9:**
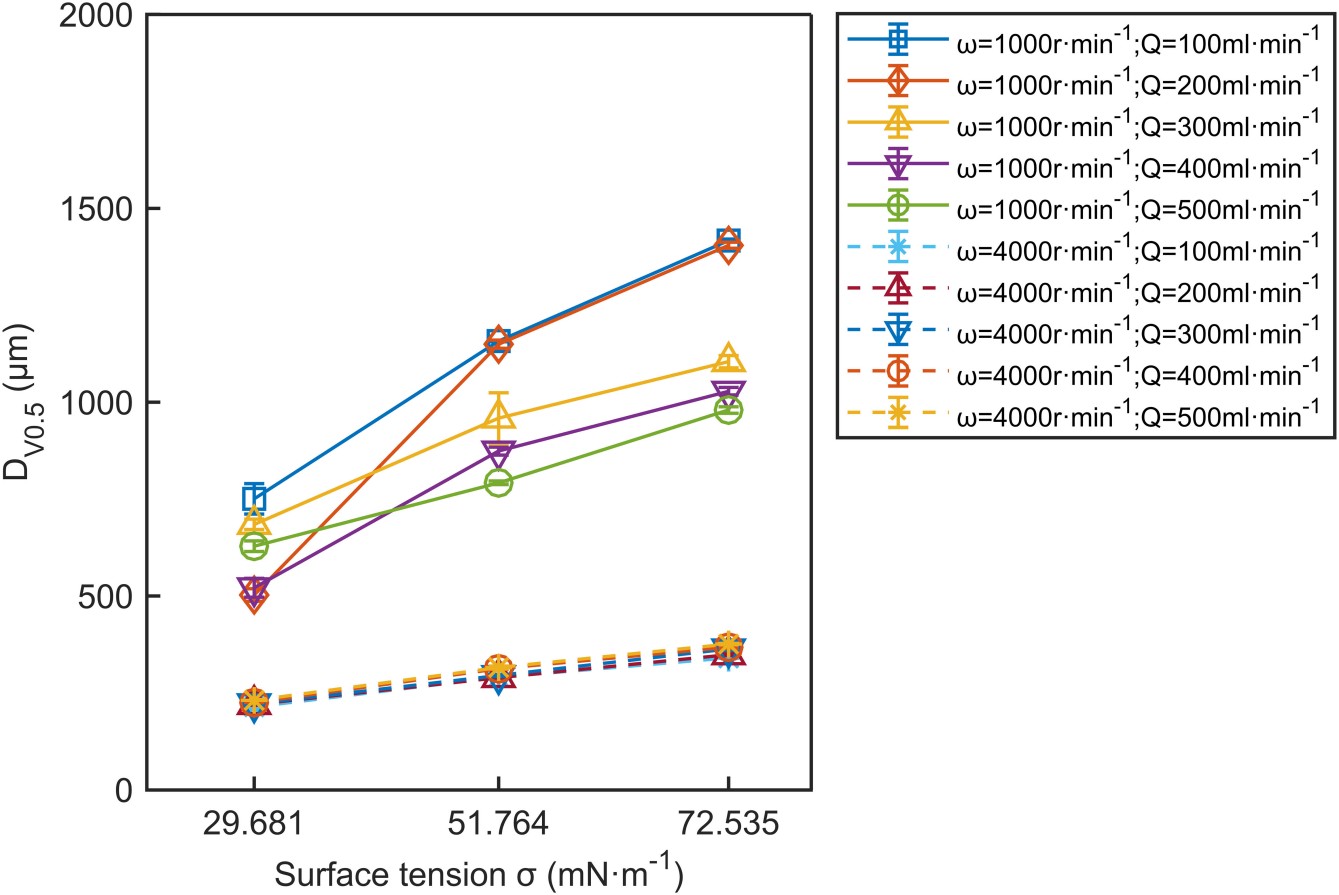
The effect of surface tension on *D*_V0.5_. The data in the figure are the average values from three replications. Error bars represent the standard errors of the means.

**Figure 9** demonstrates the significant impact of surface tension on *D*_V0.5_. Lower surface tension results in smaller droplets. The solid lines in **Figure 9** indicate that at low disc speeds, *D*_V0.5_ is strongly dependent on surface tension. In contrast, the dashed lines show that this dependence decreases significantly at high disc speeds. At high disc speeds, *D*_V0.5_ decreases as surface tension decreases. The goldenrod dashed line in **Figure 9** shows that at 4000 r·min^-1^ and a flow rate of 500 ml·min^-1^, *D*_V0.5_ decreases from 376 μm to 231 μm as surface tension drops from 72.535 mN·m^-1^ to 29.681 mN·m^-1^. This is likely because surface tension represents the force resisting the formation of new surface areas (Lefebvre and McDonell, 2017). Lower surface tension allows droplets to form more quickly, resulting in smaller droplets (Kooij et al., 2018). At low disc speeds, *D*_V0.5_ decreases significantly as surface tension decreases. The blue solid line in **Figure 9** shows that at 1000 r·min^-1^ and a flow rate of 100 ml·min^-1^, *D*_V0.5_ decreases from 1417 μm to 751 μm as surface tension drops from 72.535 mN·m^-1^ to 29.681 mN·m^-1^.

### 3.3 Droplet size distribution

**Figure 10:**
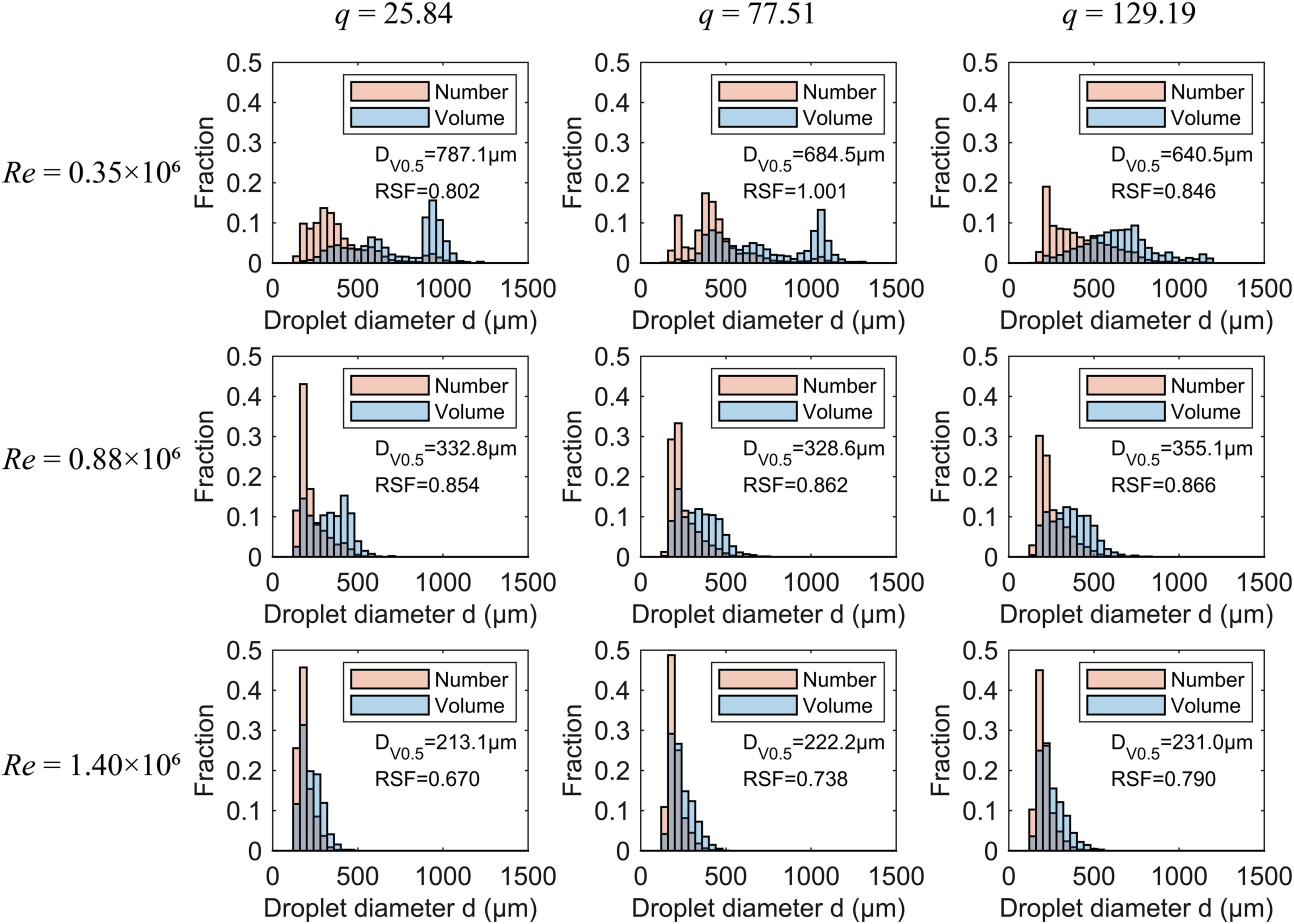
The number and volume fraction distributions for *Re* of 0.35×10^6^, 0.88×10^6^, and 1.40×10^6^, with *St* of 1.21×10^-6^ and *q* values of 25.84, 77.51, and 129.19. The orange bars represent the number fraction distribution, while the blue bars represent the volume fraction distribution.

**Figure 10** shows the distribution of droplet size in terms of both number and volume as the *Re* increases from 0.35×10^6^ to 1.40×10^6^, with *St* of 1.21×10^-6^, and *q* varying from 25.84 to 129.19. The results indicate that the droplet size distribution is positively skewed. At low *Re* and low *q*, such as *Re* = 0.35×10^6^ and *q* of 25.84 and 77.51, small droplets dominate in number but occupy only a small portion of the total volume, with most of the liquid volume concentrated in the few largest droplets. At high *Re* and high *q*, the liquid is mainly concentrated in the size range containing the most droplets. This is likely because, at higher *Re*, the liquid flow is more dominated by inertial forces, leading to flow instabilities (White and Xue, 2021) and, consequently, smaller droplet sizes. Additionally, as *Re* increases, the droplet size range narrows. For example, at *q* = 25.84 and *Re* = 0.35×10^6^, the droplet size range is within 1240 μm. When *Re* increases to 1.40×10^6^, the size range narrows further, with the maximum droplet size below 480 μm. For a given *Re*, increasing *q* from 25.84 to 129.19 slightly expands the droplet size range. Thus, the effect of *q* on droplet size distribution is less significant than that of *Re*. The RSF ranging from 0.670 to 1.001 indicates good uniformity in the droplet size distribution from the rotary atomization, as also noted by Zhou et al. (2017). Overall, *Re* has a greater impact on RSF compared to *q*. As *Re* increases, the droplet size distribution becomes more uniform. For example, at *q* = 77.51, as *Re* increases from 0.35×10^6^ to 1.40×10^6^, RSF decreases from 1.001 to 0.738. Therefore, to narrow the droplet size range and enhance uniformity, *Re* should be appropriately increased. Increasing *Re* can be achieved by enlarging the disc diameter, increasing the disc speed, raising the density, or reducing the viscosity.

**Figure 11:**
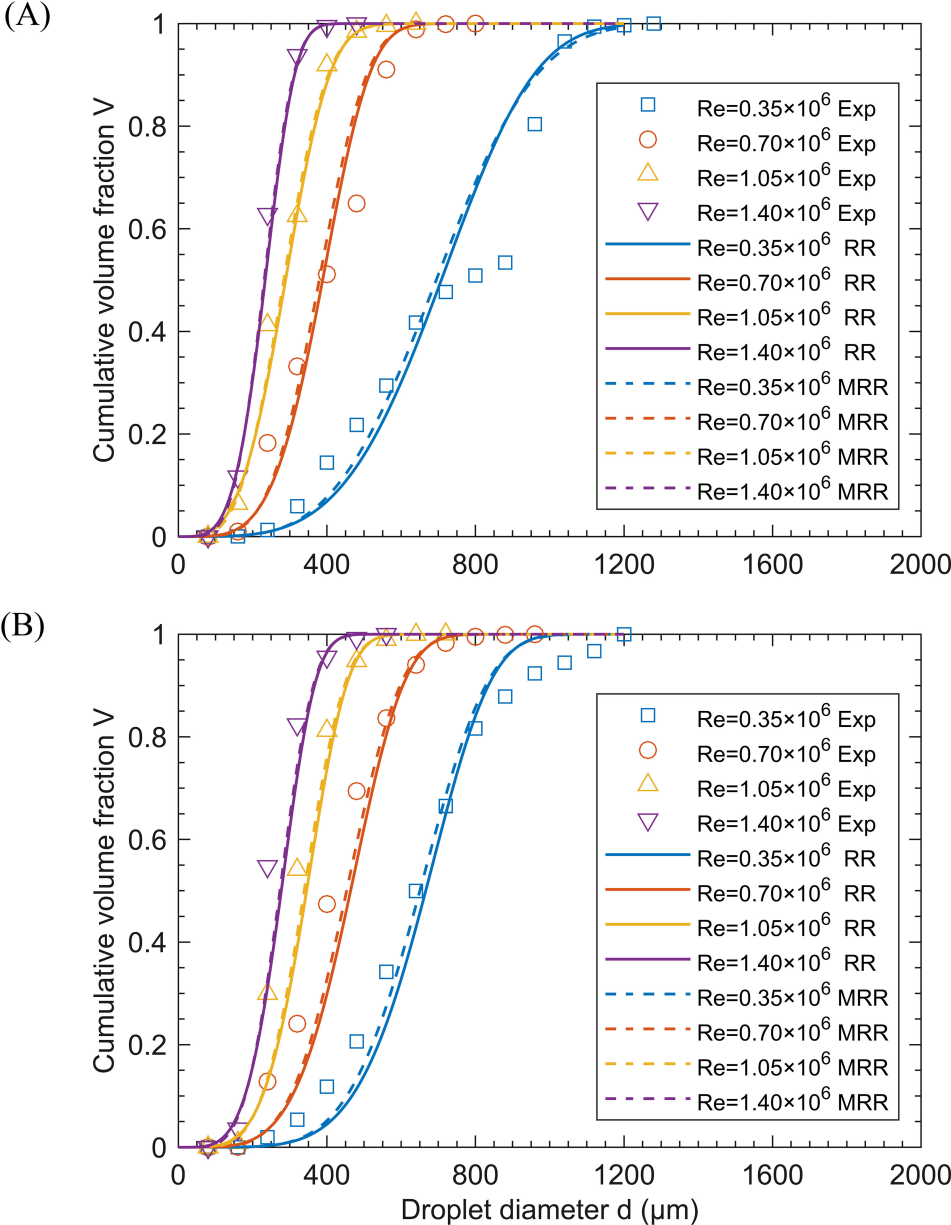
Cumulative volume fraction of droplets under different *Re* with *St* = 1.21×10^-6^. **(A)** *q* = 25.84 and **(B)** *q* = 129.19. Experimental data are compared with the fitting curves of the RR distribution (solid line) and the MRR distribution (dashed line).

**Figure 11** presents the experimental measurements and predicted cumulative volume distributions by Equations 5 and 6 at *St* = 1.21×10^-6^, with *q* values of 25.84 and 129.19, for different *Re*. As shown in the figure, as *Re* increases from 0.35×10^6^ to 1.40×10^6^, the curve becomes steeper, indicating a narrower range of droplet size distribution. This observation is consistent with the phenomenon observed in **Figure 10**. The curves in **Figure 11**, combined with the *R*² values in **Table 3**, indicate that the RR and MRR distributions can accurately describe the droplet size distribution of the spinning disc atomizer, especially at high *Re*. Additionally, the *R*² values of the MRR distribution fit are higher compared to the RR distribution. This suggests that the MRR distribution is superior to the RR distribution for spinning disc atomization with larger droplet sizes, consistent with the findings of Rizk and Lefebvre (1985). For most sprays, the RR distribution can be well described with n values between 1.5 and 4 (Lefebvre and McDonell, 2017). In **Table 3**, the n values range from 3.5 to 5.3, indicating that the spinning disc atomizer disperses droplets uniformly.

**Table 3:** Parameters of RR and MRR distribution at different *Re*, with *St* = 1.21×10^-6^ and *q* values of 25.84 and 129.19.

Distribution	*q*	Parameter	*Re* × 10^6^			
			0.35	0.70	1.05	1.40
		*n*	4.0	4.3	3.5	4.1
RR	25.84	*D*_V0.5_	708.0	388.4	288.6	233.7
		*R*²	0.948	0.948	0.977	0.984
		*n*	24.5	25.1	19.9	22.7
MRR	25.84	*D*_V0.5_	694.3	379.2	283.9	231.3
		*R*²	0.960	0.947	0.984	0.990
		*n*	5.3	4.5	4.4	4.3
RR	129.19	*D*_V0.5_	666.1	463.8	345.3	277.2
		*R*²	0.868	0.889	0.926	0.923
		*n*	32.9	27.2	25.8	24.2
MRR	129.19	*D*_V0.5_	650.4	453.0	338.9	273.5
		*R*²	0.888	0.910	0.941	0.937

## 4 Conclusions

This study used ethanol-water solutions as test liquids to experimentally investigate the atomization characteristics of the spinning disc atomizer using a high-speed camera. The effects of disc speed, flow rate, and surface tension on modes of spray formation, droplet size, and size distribution were analyzed. The main findings are as follows: (1) Under the same disc speed and ethanol-water solution, an increase in flow rate caused the modes of spray formation to transition from direct droplet formation to ligament formation, and finally to sheet formation. Frost’s transition correlations accurately predicted the spray formation modes, with the exception of ligament formation and ligament-sheet formation.

(2) It was found that the droplet sizes predicted by Frost’s correlation were significantly lower than the experimental results, with an error range of 0% to -60%. A correlation between the dimensionless droplet size d^*^ and the dimensionless quantities *Re* and *St* was established. The coefficient of determination was *R*²=0.951, and most predicted droplet sizes deviated from the experimental values by within ±15%. Under experimental conditions, *Re* and *St* were important quantities affecting droplet size. Droplet size decreased with increasing *Re* or *St*, but was hardly affected by *q*. Besides, the droplet size decreased with increasing disc speed and decreasing surface tension. At high disc speeds, the dependence of droplet size on flow rate weakened. At low disc speeds, the mode of spray formation affected droplet size, and an increase in flow rate led to a decrease in droplet size.(3) Appropriately increasing *Re* during the spinning disc atomization process can narrow the droplet size range and improve uniformity. At low *Re* and low *q*, small droplets dominate in number but occupy only a small portion of the total volume, with most of the liquid volume concentrated in the few largest droplets. At high *Re* and high *q*, the liquid is mainly concentrated in the size range containing the most droplets. The RR and MRR distributions can accurately describe the droplet size distribution, especially at high *Re*. For larger droplet sizes, the MRR distribution is superior to the RR distribution.

## Data Availability

The original contributions presented in the study are included in the article/supplementary material. Further inquiries can be directed to the corresponding authors.

## Appendix A

**Table A1:** Frost’s transition correlations and parameter ranges.

Modes of formation	Transition correlations	Parameter ranges
Droplet-Ligament	(9) $$Q<1.52\left(\frac{\sigma \rho D}{\mu^{2}}\right)\left(\frac{\mu D}{\rho }\right)/{\left(\frac{\omega \rho D^{2}}{\mu }\right)}^{0.95}$$	$\begin{array}{l} Q=60-600\text{ ml}\cdot {\text{min}}^{-1}\\ \omega =477-9549 r\cdot {\text{min}}^{-1}\\ D=0.04-0.12\text{ m}\\ \mu =0.001-0.022\text{ Pa}\cdot \text{s}\\ \rho =1000-1170\text{ kg}\cdot {\text{m}}^{-3}\\ \sigma =33-59\text{ mN}\cdot {\text{m}}^{-1} \end{array}$
Ligament	(10) $Q>0.46{\left(\frac{\sigma \rho D}{\mu^{2}}\right)}^{0.9}(\frac{\mu D}{\rho })/{\left(\frac{\omega \rho D^{2}}{\mu }\right)}^{0.63}$	
Ligament-Sheet	(11) $Q>19.8{\left(\frac{\sigma \rho D}{\mu^{2}}\right)}^{0.9}(\frac{\mu D}{\rho })/{\left(\frac{\omega \rho D^{2}}{\mu }\right)}^{0.84}$	
